# Prediction of drug target interaction based on under sampling strategy and random forest algorithm

**DOI:** 10.1371/journal.pone.0318420

**Published:** 2025-03-06

**Authors:** Feng Chen, Zhigang Zhao, Zheng Ren, Kun Lu, Yang Yu, Wenyan Wang

**Affiliations:** 1 School of Advanced Manufacturing Engineering, Hefei University, Hefei, China; 2 School of Electrical and Information Engineering, Anhui University of Technology, Ma’anshan, Anhui, China; 3 Wuhu Technology and Innovation Research Institute, AHUT, Wuhu, China; The First Hospital of Jilin University, CHINA

## Abstract

Drug target interactions (DTIs) play a crucial role in drug discovery and development. The prediction of DTIs based on computational method can effectively assist the experimental techniques for DTIs identification, which are time-consuming and expensive. However, the current computational models suffer from low accuracy and high false positive rate in the prediction of DTIs, especially for datasets with extremely unbalanced sample categories. To accurately identify the interaction between drugs and target proteins, a variety of descriptors that fully show the characteristic information of drugs and targets are extracted and applied to the integrated method random forest (RF) in this work. Here, the random projection method is adopted to reduce the feature dimension such that simplify the model calculation. In addition, to balance the number of samples in different categories, a down sampling method NearMiss (NM) which can control the number of samples is used. Based on the gold standard datasets (nuclear receptors, ion channel, GPCRs and enzymes), the proposed method achieves the auROC of 92.26%, 98.21%, 97.65%, 99.33%, respectively. The experimental results show that the proposed method yields significantly higher performance than that of state-of-the-art methods in predicting drug target interaction.

## 1. Introduction

The prediction of drug-target interaction (DTI) at the molecular level is an effective way to promote the development of drug discovery and drug repositioning [1–3]. As a compound with chemical structure characteristics, drugs play a significant role in human body by interacting with one or more targets. Proteins is the representative type of target, and their functions can be inhibited, enhanced or blocked by drugs to achieve the treatment and prevention of various diseases [4]. Currently, biomedical experimental wet-lab techniques and computational methods are two main approaches to obtain the interaction information of drug and targets. However, the former determination of DTIs is both time- and resource-consuming, which restricts the development and repurposing of drugs. Moreover, it has a high failure rate and blindness due to the lack of prior knowledge of DTI. Therefore, it is necessary to develop efficient computational methods to assist in the prediction of drug target interactions.

In recent decades, many in silico approaches have been developed for the prediction of new drug-target interactions, and ligand-, target- and chemo-genomic-based are three most commonly used methods among them [5,6]. With the accessibility of big sources such as genome, phenome, drug chemical structures, biological bioassays and interaction group, the diversity of information related to drug (chemical space) and target (genomic feature space) that can be extracted is gradually increasing. And therefore the traditional methods include the ligand- and target-based approaches can be very challenging to deal with flexibility target proteins. Therefore, the research of chemo-genomic methods is highly attractive to make full use of the heterogeneous biological data of known DTIs [7,8].

Predicting DTIs using machine learning is one of the main means to realize chemo-genome prediction. In these methods, the problem of DTI prediction is transformed into a binary task to identify whether there is interaction between drugs and targets [9]. However, in the database of DTIs, the number of drug target pairs with identified interactions is relatively small. In other words, there is a quantitative imbalance between different sample categories. To solve this problem, Rayhan et al. proposed a novel modified cluster based under sampling method, and achieved the ROC (operating characteristic curve) on the four sub datasets (enzymes, ion channels, GPCRs, and nuclear receptors) of the gold standard dataset are 96.89%, 93.69%, 93.22% and 92.85% respectively [10]. Mousavian et al. applied the random sampling method to the set with a large number of negative samples to balance the data set [11]. Liu et al. took the compound–protein pairs that locate far from all positive samples in the chemogenomical space as negative samples, and the results show that this negative sample screening method is helpful to improve the performance of classical classifiers and existing computing methods [12]. In addition, synthetic over sampling, balanced random sampling, neighborhood cleaning rule have been employed to balance the imbalanced datasets, etc. [13–17].

Currently, benefit from the diversified expression of digital features, the feature vector-based methods have shown great potential in improving the prediction performance of DTI, in which the feature can be defined as the combination of one or more drug descriptors and one or more target descriptors. Hu et al. calculated drug descriptors by PaDEL-Descriptors, and extracted 115 properties of each target from AAindex1 database. After applying the 2,916 dimensional eigenvectors to DTI prediction, the AUC of 99.66% was obtained on the test set [18]. Bahi et al. predicted the interactions combined with 193-dimensional drug descriptors and 1290-dimensional target-descriptors [19]. Wei et al. used 881 dimensional drug molecular fingerprints extracted from PubChem and 567 and 1,449 dimensional protein sequence characteristic to predict the DTIs [20]. Generally, the larger the dimension of the feature vector in the data set, the greater the possibility of obtaining an effective model, but the amount of model calculation will also increase.

In addition, graph neural networks have also been applied to DTI prediction and its related works. For example, Hu et al. **e**stablished an improved graph representation learning method, namely iGRLDTI, to address the over-smoothing simulation issue by better capturing more discriminative representations of drugs and targets in a latent feature space [21]. Zhao et al. proposed a novel graph representation learning model, namely FuHLDR, for drug repositioning and they also developed a deep learning framework, namely DDAGDL, to predict drug-drug associations (DDAs) by using geometric deep learning (GDL) over heterogeneous information network [22,23]. Furthermore, in order to investigate the underlying network structure of the graph for better representation of drug and target information, a new fuzzy based deep AG clustering model has been developed to explore the key dependency relationships between node embeddings and result clusters [24,25].

In this work, to comprehensively express the drug and target information, 10 kinds of molecular fingerprint information and their related counting vectors, a total of 12 kinds of drug feature descriptors, were extracted. Moreover, the six amino acid sequence characteristics of target proteins were selected. In the process of data processing, a random projection dimensionality reduction method is firstly used to reduce the huge amount of computation brought by multi-dimensional features to the model. Then, to reduce the performance impact of excessive negative samples with non-interaction between drug targets, Near Miss method is used for sampling negative samples. Finally, the balanced data set after dimensionality reduction is input into the random forest model. As a result, the DTI prediction method proposed in this work achieves the state-of-the-art performance on the four sub-datasets of the gold standard data set.

In summary, the main contributions of this work are as follows:

- To describe drug and target information, 10 types of molecular fingerprint information and their related counting vectors were used to extract drug features and 6 target features for predicting drug target interactions.
- A suitable combination of downsampling, dimensionality reduction, and classifier was found for predicting drug target interactions on imbalanced datasets.
- The simple method proposed in this work has achieved good classification performance on Enzyme, GPCR, Ion channel, and Nuclear receptor datasets.

## 2. Materials and methods

**Fig 1:**
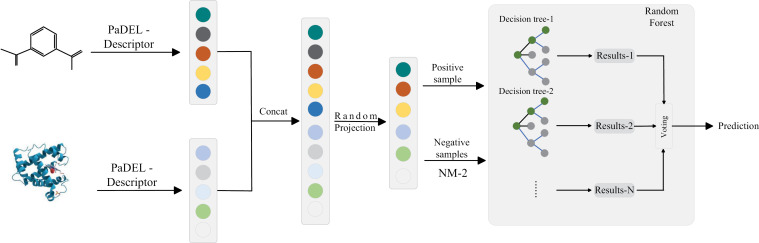
The overall framework of the model proposed in our work.

The overall architecture of the drug-target interaction prediction model proposed in this work is shown in Fig 1. Our proposed model first concatenates the fingerprint and statistical features of the extracted drugs and targets. Secondly, the random projection method is used to remove redundant features and reduce the computational complexity. Then, the NearMiss method is used to balance the positive and negative samples in the dataset. Finally, the random forest classifier was used for predicting drug target interactions. More details of our model are described below.

### 2.1. Benchmark datasets

The data used in this work is come from the Gold Standard Dataset, which was first introduced by Yamanishi et al. in 2008 and have been used by researchers in recent years [26–28]. It collects and constructs the DTI information from KEGG, DrugBank, BRENDA, and SuperTarget [29–32]. According to different types of target proteins, the database was separated and named into four sub datasets: the enzyme, GPCR (G-protein-coupled receptors), ion channel, and nuclear receptor. All researchers are available from public websites at http://web.kuicr.kyoto-u.ac.jp/supp/yoshi/drugtarget/. Table 1 shows the information of drugs, targets and their interactions in the four sub data sets. It can be noted that the number of positive samples of known drug target interaction is much smaller than that of negative samples. In other words, these four data sets are seriously unbalanced.

**Table 1:** The information of drugs, targets and their interactions in the four sub data sets.

Data sets	Known interactions	Unknown interactions	Drugs	Targets
Enzyme	2,926	292,554	445	664
GPCR	635	20,550	223	95
Ion channel	1,476	41,364	210	204
Nuclear receptor	90	1,314	54	26

To better predict the drug target interaction, many feature descriptors of drugs and targets are extracted. Specifically, 797 descriptors and 10 molecular fingerprint features based on multiple structural forms such as MOL and SMILES of drugs, were extracted by the PaDEL-Descriptor software. In addition, six target feature descriptors were selected based on the amino acid sequence of protein. The specific features of drug and target proteins are listed in Table 2. It can be seen that there are 17,740 features for a drug target pair [33,34].

**Table 2:** List of drug- and target-descriptors.

Drug-descriptors name	Dimension	Drug-descriptors name	Dimension
Atom Pairs 2D Fingerprint Count (Atom-Count)	780	Klekota Roth Fingerprint Count (Klek-Count)	4,860
Atom Pairs 2D Fingerprint (Atom-Fingerprints)	780	Klekota Roth Fingerprint (Klek-Fingerprints)	4,860
Electrotopological State Fingerprint (Estate-Fingerprints)	79	MACCS Fingerprint	166
Extended Fingerprint	1,024	PubChem Fingerprint	881
Fingerprint	1,024	Substructure Fingerprint Count (Sub-Count)	307
Graph Only Fingerprint (Graph-Only)	1,024	Substructure Fingerprint (Sub-Fingerprints)	307
**Target-descriptors name**	**Dimension**	**Target-descriptors name**	**Dimension**
Amino Acid Composition (AAC)	20	Composition, Transition, Distribution (CTD)	504
Total Amino Acid Properties (AAP)	484	Dipeptide Composition (DPC)	400
Amphiphilic Pseudo-amino acid composition (APAAC)	80	Quasi-sequence-order descriptors (QSO)	160

### 2.2. Evaluation criteria

A variety of evaluation indicators can be used to show and compare the classification performance of the model, such as accuracy and precision. However, only using these indicators is of little significance for the evaluation of unbalanced datasets. Following the previous studies, the area under the curve for the receiver operating characteristic (Area Under ROC, AUC) is used as an performance criteria in this work. For each prediction model, some evaluation parameters can be calculated as follows:


$$Accuracy=\frac{TP+TN}{TP+TN+FP+FN}$$ (1)

$$Precision=\frac{TP}{TP+FP}$$ (2)

$$Recall=\frac{TP}{TP+FN}$$ (3)

$$F1\_score=\frac{2\times Precision\times Recall}{Precision+Recall}$$ (4)

where *TP, TN, FP, FN* are true positive, true negative, false positive and false negative, respectively. In this work, positive refer to the interaction between drugs and targets, otherwise, they are negative cases. *TP* indicates that the interacting drugs and targets are correctly predicted. Conversely, *FP* represents that the drug target with non-interaction is incorrectly predicted as a positive sample. Precision-recall curve (PR curve) can be drawn based on different recall and precision, and the receiver operating characteristic curve (ROC curve) can be obtained based on different recall and false positive rates. auROC and auPR are the area under the ROC and PR curve, respectively.

### 2.3. Random projection and negative sampling

#### 2.3.1. Random projection.

The multimodal characteristics of drugs and targets improve the sample expression ability. While the high-dimensional of these features increase the amount of calculation of the model, and there may be information redundancy between them. Therefore, to speed up the computations of machine learning task, a data compression method based on random projection, which can project high-dimensional data into low-dimensional subspace, is adopted in this work.

Random projection is an approximate algorithm for estimating distances between pairs of samples in a high-dimensional vector space [35,36]. Given a training dataset $X\in R^{N\times L_{1}}$, which contains *N* samples with $L_{1}$dimensions. The random projection method multiplies *X* by a random matrix $R\in R^{L_{1}\times L_{2}}$. Typically, R consists of entries of standard normal $N\left(0,1\right)$. However, it has been proved that sparse random projections can achieve three times the acceleration in processing time by replacing the $N\left(0,1\right)$ entries in R with entries in $\left\{-1,0,1\right\}$ with probabilities $\left\{\frac{1}{6},\frac{2}{3},\frac{1}{6}\right\}$. Therefore, in this work, sparse random projections is adapted. The calculation formula is as follows:


$$X^{R}=XR=\sum_{i}x_{i}r_{i}$$ (5)

where $X^{R}\in R^{N\times L_{2}}$, $x_{i}$ is the data of the *i*th sample, $r_{i}$ is the *i*th column of the random matrix and $L_{1}\ll L_{2}$. In this work, $L_{1}$ and $L_{2}$ are 17,740 and 8,870, respectively.

#### 2.3.2. Negative sampling.

Data imbalance is the common problem in machine learning, especially for medical datasets [37,38]. Under-, over- or mixed sampling are three typical techniques to solve this issue [39,40]. However, for the prediction of DTI, it is impractical to reduce this difference by generating new false samples when over-sampling method is used, because these drugs and/or targets are not exist in the real world.

**Fig 2:**
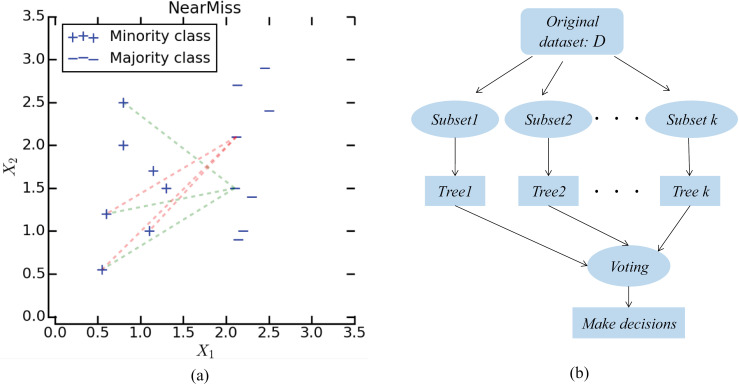
(a) The schematic diagram of negative sample sampling; (b) The framework of the random forest classification algorithm.

Currently, instance hardness threshold (IHT), random under sampler, neighborhood cleaning rule and cluster centroids, as down sampling methods, have been widely used to filter samples for categories with large sample size [41]. Among them, NearMiss, which can control the number of selected samples, has attracted great attention in biomedical research in recent years. To alleviate the problem of information loss in random under sampling, it selects the most representative samples from the majority class for which the average distance to the farthest neighbors is the smallest. Fig 2(a) shows a schematic diagram of negative sample sampling. In this work, the number of neighbors is set to 3.

### 2.4. Random forest classifier

Random forest as an ensemble classifier based on decision tree has been widely used in classification and regression tasks [42]. Its purpose is to coordinate the results of multiple weak classifiers, so as to obtain more accurate classification performance [43,44]. Given a data set *D*, the random forest first randomly puts back the samples for multiple times to generate *k* sample subsets $D_{1},D_{2},\ldots ,D_{k}$,; Then, these subsets are fed into different base decision trees (models), $M_{1},M_{2},\ldots ,M_{K}$*,* to produce weak prediction results. In this work, the drug target interaction prediction problem is transformed into a binary classification task, and therefore, the final result $H\left(x\right)$ of RF$M^{*}$is generated by multiple weak classification votes $h_{i}\left(x\right)$ [45,46]. The framework of the random forest classification algorithm is shown in Fig 2(b).

In order to ensure the diversity of base classifiers in the RF classifier, the sampling method is not only used to select samples, but also adapted to sample features to control the growth shape of the tree. The differences between these various base models improve the generalization ability and avoid over fitting of the model $M^{*}$ [47]. Therefore, all experiments in this work were performed five times to avoid the random error.

## 3. Results

### 3.1. Performance on DTIs

To avoid over fitting or instability of our model caused by a small number of samples in the datasets, such as nuclear receptors dataset only containing 180 samples, 10-fold cross-validation is adapted in this work, and therefore the final result of each experiment is the average of the prediction results. Meanwhile, all experiments were performed five times to ensure the robustness and effectiveness of the method. Table 3 lists the average and standard deviation of several experimental results on four datasets.

**Table 3:** The prediction performance of five times 10-fold cross-validation on four datasets generated by using the proposed method.

	Enzyme	Ion channels	GPCR	Nuclear receptor
Accuracy (%)	99.57 ± 0.85	97.22 ± 0.51	98.03 ± 0.51	91.67 ± 2.59
Recall (%)	99.15 ± 1.00	96.68 ± 0.66	96.78 ± 1.10	87.49 ± 2.48
Precision (%)	99.98 ± 0.77	97.76 ± 0.79	99.37 ± 0.26	96.39 ± 3.53
F1-scores (%)	98.57 ± 0.86	97.21 ± 0.51	98.05 ± 0.60	91.34 ± 2.56
auROC (%)	**99.33** ± 0.02	**98.21** ± 0.14	**97.65** ± 0.56	**92.26** ± 1.94
auPR (%)	**99.73** ± 0.17	**98.87** ± 0.10	**98.63** ± 0.33	**94.51** ± 1.39

It can be seen from Table 3 that our proposed method can achieve good prediction results on four datasets, especially for Enzyme and Ion channels, which the auROC values can reach as much as 99% and 98%. More specifically, on the Enzyme dataset, it yielded an accuracy of 99.57%, precision of 99.98%, recall of 99.15%, f1-score of 98.57%, auROC of 99.33% and auPR of 99.73%. And their standard deviations are 0.85, 1.00, 0.77, 0.86, 0.02 and 0.17. When the ion channels dataset is used to predict DTIs, the average accuracy, recall, precision, f1-score auROC and auPR are 97.22%, 96.68%, 97.76%, 97.21%, 98.21% and 99.87%, respectively. The standard deviations of them are 0.51, 0.66, 0.79, 0.51, 0.14 and 0.10. which shows that the prediction performance of our model is robust and stable. Similarly, our model is also implemented on GPCR and nuclear receptor data sets, and auPRs of 98.63% and 98.87% are obtained respectively, which represents that the method proposed in this work has satisfactory generalization ability.

However, it is worth noting that although our model achieved satisfactory classification performance on all four datasets, comparing the classification performance between different datasets, we found that the auROC of the Nuclear receptor, GPCR, Ion channels, and Enzyme datasets were 92.26%, 97.65%, 98.21%, and 99.33%, respectively, indicating an increasing trend in their performance. We further compared the results with Table 1 and found that their interacting target pairs were 90, 635, 1,476 and 2,926, respectively. Based on this, we speculate that the classification performance on different datasets may be related to the number of drug target pairs in the dataset, and we believe this inference is reasonable because the size of the dataset affects the learning ability of the model. When the dataset is small, the model does not learn sufficiently, and therefore leading to lower performance.

### 3.2. Comparisons with other state-of-the-art methods

Recently, many methods have achieved great success in the drug target interactions prediction. To evaluate the effectiveness of our proposed method, several state-of-the-art methods are compared on auROC values when the same Gold Standard Dataset is used. Table 4 shows the performance of different prediction methods. It can be seen that the most of these methods have the auROC values greater than 0.9, which indicates a good effect in the prediction of DTI.

**Table 4:** Comparison of auROC values with several state-of-the-art methods.

Method	Enzyme	GPCR	Ion channels	Nuclear receptor
PUDT [48]	0.884	0.878	0.831	0.885
Wang et al. [49]	0.943	0.874	0.911	0.818
MDFR [20]	0.969	0.904	0.933	0.886
Cao et al. [50]	0.949	0.890	0.943	0.882
FRnet-DTI [27]	0.975	0.948	0.951	0.924
Ensemble-MFP[51]	0.959	0.943	0.960	0.939
SRX-DTI [48]	0.992	0.978	0.988	0.932
**Our method**	**0.993**	**0.981**	**0.985**	**0.930**

For the prediction of the DTI, the potential influential features are extracted from different perspectives. Lan et al. set unknown interactions as unlabeled samples and proposed a method called PUDT to predict drug target interactions. On the same datasets in this work, PUDT achieved classification results of 0.884, 0.878, 0.831, and 0.885, respectively [48]. Wang et al. proposed a stacked auto-encoder of deep learning to mine the hidden information in protein sequences, and then combined them with molecular fingerprint information to accurately predict DTI [49]. It can be found that the auROC of 94.3% on Enzyme, 87.4% on GPCR, 91.1% on Ion channels, and 81.8% on nuclear receptor can be obtained, which shows that this method is effective in predicting the interaction. Similarity, to convert high-dimensional features to low-dimensional features, a multi-scale features deep representations inferring interactions (MDFR) method is proposed, which also uses an auto-encoder to reconstruct drug and protein features [20]. The results show that the multi-scale feature is more effective in predicting the interaction. Cao et al. uses structural and physicochemical properties simultaneously from drugs and proteins for interaction prediction [50]. FRnet-DTI propose two convolution neural network, including FRnet-Encode and FRnet- predict. One model is used for feature manipulation and the other one for classification [27]. Based on the negative sample sampling method of Euclidean distance, the Ensemble-MFP (Ensemble model of Multiple Feature Pairs) model is proposed to predict DTIs [51]. As a competitive DTI method, SRX-DTI explored the impact of different feature combinations on model performance and adopted similar feature dimensionality reduction and sampling methods similar to our work. As a result, it achieved excellent performance of over 0.9 on four datasets [48]. Better yet, in this work, we not only consider more comprehensive drug target information, but also the influence of calculation dimension and data imbalance, and the experiments demonstrated that our proposed method is more effective in predicting DTIs.

## 4. Discussion

### 4.1. Comparison of classification performance with different reduced dimensions and stochastic projection matrix

Feature dimensionality reduction can effectively reduce the computational cost of the model. To investigate the influence of the selection of random projection matrices such as Gaussian random matrix and sparse random matrix, as well as their dimensionality reduction parameter L2, on the predictive performance of the model, this work conducted experiments with 10% dimensionality reduction each time to find the optimal balance between computational cost and performance, and further discussed in detail the impact of the selection of random projection matrices. S1 and S2 Tables show the predictive performance of the proposed method under different feature dimensions when selecting Gaussian random matrix and sparse random matrix, respectively.

It can be observed from S1 Table that the performance of our model for DTI prediction is affected by features with different dimensions, and their impact on the evaluation indicators does not increase or decrease regularly with the increase of feature dimension, which indicates that there is information redundancy in feature descriptors of drug and target protein. However, the overall F1 value of the comprehensive indicator of the model did not show significant fluctuations, indicating the comprehensiveness of the features used in this work and the stability of our proposed method. In addition, this work further explores the impact of different L2 values on model performance when using sparse random matrices, as shown as S2 Table. It shows the improvement of model performance with increasing dimensionality. However, this phenomenon is also not deterministic. Ideally, the model performance achieves a good balance between performance and dimensionality at a dimension of 7,096. However, comparing sparse random matrix with gaussian random matrix, it can be found that on the nuclear receptor dataset, the use of gaussian random matrix method results in 2% and 3% performance improvement in auROC and auPR, respectively. Therefore, the final method used in this work is gaussian random projection, with an L2 value of 8,870.

###  4.2. Comparison of different sampling methods

**Fig 3:**
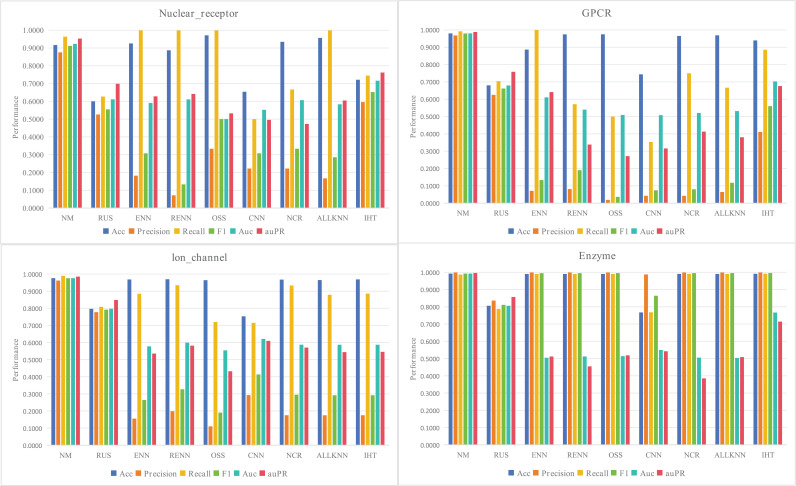
Performance comparison of different sampling methods.

As mentioned earlier in the methods section, there exists a notable imbalance in the proportion of drug and targets that interact with each other within drug target interaction pairs. This imbalance poses a challenge for prediction models, as they may achieve high overall accuracy but perform poorly in precision predicting interactions. To address the issue of high false negatives in the prediction results, this study investigates the effectiveness of various sampling methods for DTIs. Specifically, different under-sampling methods, including near miss (NM), edited nearest neighbours (ENN), repeated edited nearest neighbours (RENN), ALLKNN, random under sampler (RUS), condensed nearest neighbour (CNN), one sided selection (OSS), neighbourhood cleaning rule (NCR), and instance hardness threshold (IHT), are compared. The performance of these methods is evaluated on four datasets, and the results are presented in Fig 3. The findings indicate that the NM method outperforms others in terms of all metrics, making it the most suitable technique for selecting negative samples in this study.

### 4.3. Comparison between different classifiers

**Fig 4:**
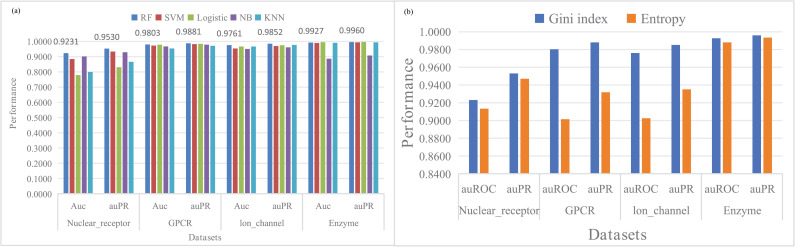
(a) Performance comparison between different classifiers. (b) Performance comparison between different splitting criteria in random forests.

Support vector machine is a supervised learning algorithm, which has been proved to have outstanding classification performance in the task of predicting drug target interaction. In this section, the prediction performance of random forest, support vector machine and other classifier are compared when the same feature is used. The comparison results are shown in the Fig 4. It can be seen that on the data sets Nuclear receptor, GPCR, Ion channels and Enzyme, the auROCs obtained by the support vector machine classifier are 92.31%, 98.03%, 97.61% and 99.27%, and auPR values of these datasets are 95.30%, 98.81%, 98.52% and 99.60% respectively. In contrast, the random forest algorithm has achieved good performance in both auROC and auPR metrics, indicating its effectiveness in this work.

### 4.4. Comparison of different parameters of random forest classifier

In addition to experimental verification of sampling and dimensionality reduction methods for each module mentioned above, this work also conducted detailed experimental verification on the tree depth and splitting criterion of the basic classifier in random forests, and the results showed that different parameters will affect the model’s predictive performance in different ways. The detailed information is shown in S3 and S4 Tables and Fig 4(b). It can be observed from S3 Table that the classification performance of the model continuously improves with the increase of the number of trees, while the running time of the model also increases. Similarly, basic classifiers that use Gini index as the splitting rule for trees always achieve better classification performance than selection entropy. Specifically, the impact of tree depth on performance is irregular. As shown in S4 Table, taking the Nuclear receptor dataset as an example and keeping other parameters fixed, when the tree depths are 10 and 20, the model’s auROC is 88.87% and 89.68%, respectively. When the tree depth deepens to 30, the auROC is 88.84% as shown in Fig 4(b). As a result, the optimal classifier parameters are 1,000 trees, Gini index as the splitting criterion, and unrestricted tree growth depth.

## 5. Conclusion

In this work, a novel computational method combining multiple drug molecular fingerprints and protein sequence information is proposed for drug target interaction prediction. To extract more representative features and reduce the calculation, we use a random projection that does not change the distance between samples to greatly cut off the original feature dimension. And then a down sampling method called NearMiss which can control the number of filtered samples is adopted to avoid the high false positive rate caused by data imbalance. Finally, the new balanced sample set and features are fed into the random forest classifier. Experimental results show that our method proposed in this work has the best performance and generalization ability on four data sets nuclear receptors, ion channel, GPCRs and enzymes.

## Supporting information

S1 Table Performance of our proposed model with different feature dimensions when using gaussian random matrix. (DOCX)

S2 Table Performance of our proposed model with different feature dimensions when using sparse random matrix. (DOCX)

S3 Table The impact of different tree depths on model performance in random forests. (DOCX)

S4 Table The impact of the number of trees in a random forest on model performance. (DOCX)
