# Correction: Prediction of drug target interaction based on under sampling strategy and random forest algorithm

**DOI:** 10.1371/journal.pone.0330276

**Published:** 2025-08-12

**Authors:** Feng Chen, Zhigang Zhao, Zheng Ren, Kun Lu, Yang Yu, Wenyan Wang

There are errors in the author affiliations. The correct affiliations are as follows:

Feng Chen^1^, Zhigang Zhao^2^, Zheng Ren^2^, Kun Lu^2^, YangYu^3^, Wenyan Wang^2,4^

**1** Institute of Embodied Intelligence, Anhui University, Hefei, Anhui, China, **2** School of Electrical and Information Engineering, Anhui University of Technology, Ma’anshan, Anhui, China, **3** School of Advanced Manufacturing Engineering, Hefei University, Hefei, China, **4** Wuhu Technology and Innovation Research Institute, AHUT, Wuhu, China
