# Supplementary material for: Prediction of drug target interaction based on under sampling strategy and random forest algorithm
Source: PLoS One. 2025 Mar 6;20(3):e0318420. doi: 10.1371/journal.pone.0318420 (PMC11884685; doi:10.1371/journal.pone.0318420)
Supplement: S1 Table — (DOCX) [file pone.0318420.s001.docx]

| **Dataset** | **Metrics** | **Dimensions** | | | | | | | | | |
| --- | --- | --- | --- | --- | --- | --- | --- | --- | --- | --- | --- |
|  |  | 17,740 | 15,966 | 14,192 | 12,418 | 10,644 | **8,870** | 7,096 | 5,322 | 3,548 | 1,774 |
| Nuclear_receptor | acc | 0.9000 | 0.9111 | 0.9000 | 0.8833 | 0.9000 | 0.9167 | 0.9222 | 0.8944 | 0.8889 | 0.8778 |
|  | pre | 0.8692 | 0.8900 | 0.8894 | 0.8706 | 0.8674 | 0.8755 | 0.8977 | 0.8769 | 0.8880 | 0.9088 |
|  | rec | 0.9371 | 0.9385 | 0.9232 | 0.9085 | 0.9389 | 0.9639 | 0.9478 | 0.9187 | 0.8949 | 0.8640 |
|  | F1 | 0.8939 | 0.9090 | 0.8970 | 0.8818 | 0.8944 | 0.9118 | 0.9183 | 0.8880 | 0.8850 | 0.8803 |
|  | auROC | 0.9112 | 0.9192 | 0.9119 | 0.8948 | 0.9097 | **0.9231** | 0.9296 | 0.9016 | 0.8989 | 0.8770 |
|  | auPR | 0.9393 | 0.9448 | 0.9369 | 0.9256 | 0.9393 | **0.9530** | 0.9506 | 0.9311 | 0.9220 | 0.9114 |
| GPCR | acc | 0.9717 | 0.9803 | 0.9772 | 0.9811 | 0.9803 | 0.9803 | 0.9646 | 0.9740 | 0.9756 | 0.9717 |
|  | pre | 0.9608 | 0.9654 | 0.9641 | 0.9701 | 0.9654 | 0.9684 | 0.9623 | 0.9561 | 0.9668 | 0.9590 |
|  | rec | 0.9827 | 0.9952 | 0.9904 | 0.9919 | 0.9949 | 0.9921 | 0.9672 | 0.9920 | 0.9837 | 0.9839 |
|  | F1 | 0.9714 | 0.9800 | 0.9768 | 0.9808 | 0.9798 | 0.9799 | 0.9646 | 0.9734 | 0.9757 | 0.9711 |
|  | auROC | 0.9716 | 0.9803 | 0.9774 | 0.9811 | 0.9805 | **0.9803** | 0.9645 | 0.9742 | 0.9757 | 0.9717 |
|  | auPR | 0.9816 | 0.9890 | 0.9863 | 0.9885 | 0.9888 | **0.9881** | 0.9742 | 0.9851 | 0.9835 | 0.9817 |
| lon_channel | acc | 0.9777 | 0.9709 | 0.9665 | 0.9763 | 0.9668 | 0.9722 | 0.9705 | 0.9736 | 0.9682 | 0.975 |
|  | pre | 0.9837 | 0.9735 | 0.9676 | 0.9851 | 0.9729 | 0.9776 | 0.9817 | 0.9728 | 0.973 | 0.983 |
|  | rec | 0.9717 | 0.9679 | 0.9644 | 0.9678 | 0.9608 | 0.9668 | 0.96 | 0.9741 | 0.9635 | 0.9674 |
|  | F1 | 0.9776 | 0.9707 | 0.9659 | 0.9763 | 0.9668 | 0.9721 | 0.9707 | 0.9733 | 0.9681 | 0.9751 |
|  | auROC | 0.9846 | 0.9846 | 0.9777 | 0.9776 | 0.9703 | **0.9761** | 0.9684 | 0.9734 | 0.9825 | 0.9598 |
|  | auPR | 0.9895 | 0.9895 | 0.9857 | 0.9841 | 0.9776 | **0.9852** | 0.9802 | 0.9806 | 0.9897 | 0.9722 |
| Enzyme | acc | 0.9952 | 0.9935 | 0.9952 | 0.9948 | 0.9938 | 0.9957 | 0.9966 | 0.9925 | 0.9902 | 0.9878 |
|  | pre | 0.9997 | 0.999 | 0.9996 | 0.9993 | 0.9976 | 0.9998 | 0.9997 | 0.9987 | 0.9989 | 0.9935 |
|  | rec | 0.9904 | 0.9881 | 0.9907 | 0.9892 | 0.9898 | 0.9915 | 0.9935 | 0.9865 | 0.9817 | 0.9823 |
|  | F1 | 0.9976 | 0.9935 | 0.9951 | 0.9946 | 0.9937 | 0.9957 | 0.9966 | 0.9926 | 0.9902 | 0.9878 |
|  | auroc | 0.9935 | 0.9951 | 0.9951 | 0.9931 | 0.9965 | **0.9927** | 0.9930 | 0.9945 | 0.9908 | 0.9918 |
|  | auPR | 0.9965 | 0.9974 | 0.9975 | 0.9961 | 0.9982 | **0.9960** | 0.9961 | 0.9973 | 0.9948 | 0.9949 |
