# Supplementary material for: Prediction of drug target interaction based on under sampling strategy and random forest algorithm
Source: PLoS One. 2025 Mar 6;20(3):e0318420. doi: 10.1371/journal.pone.0318420 (PMC11884685; doi:10.1371/journal.pone.0318420)
Supplement: S2 Table — (DOCX) [file pone.0318420.s002.docx]

| **Dataset** | **Metrics** | **Dimensions** | | | | | | | | | |
| --- | --- | --- | --- | --- | --- | --- | --- | --- | --- | --- | --- |
|  |  | 17,740 | 15,966 | 14,192 | 12,418 | 10,644 | 8,870 | **7,096** | 5,322 | 3,548 | 1,774 |
| Nuclear_receptor | acc | 0.8889 | 0.8778 | 0.8889 | 0.8556 | 0.8944 | 0.8778 | 0.8889 | 0.9056 | 0.8889 | 0.8611 |
|  | pre | 0.8470 | 0.8692 | 0.8640 | 0.8874 | 0.8775 | 0.8692 | 0.8817 | 0.9134 | 0.8561 | 0.8324 |
|  | rec | 0.9389 | 0.8990 | 0.9145 | 0.8381 | 0.9145 | 0.9035 | 0.9088 | 0.8967 | 0.9357 | 0.8965 |
|  | F1 | 0.8788 | 0.8724 | 0.8847 | 0.8563 | 0.8890 | 0.8772 | 0.8850 | 0.9009 | 0.8855 | 0.8524 |
|  | auROC | 0.9005 | 0.8913 | 0.8972 | 0.8649 | 0.9038 | 0.8852 | **0.9024** | 0.9126 | 0.8977 | 0.8715 |
|  | auPR | 0.9346 | 0.9202 | 0.9254 | 0.8933 | 0.9293 | 0.9225 | **0.9286** | 0.9272 | 0.9348 | 0.9089 |
| GPCR | acc | 0.9732 | 0.9772 | 0.9780 | 0.9717 | 0.9780 | 0.9780 | 0.9811 | 0.9717 | 0.9795 | 0.9622 |
|  | pre | 0.9606 | 0.9671 | 0.9670 | 0.9574 | 0.9638 | 0.9654 | 0.9687 | 0.9594 | 0.9669 | 0.9431 |
|  | rec | 0.9859 | 0.9872 | 0.9890 | 0.9857 | 0.9918 | 0.9904 | 0.9936 | 0.9838 | 0.9922 | 0.9802 |
|  | F1 | 0.9728 | 0.9769 | 0.9777 | 0.9712 | 0.9775 | 0.9777 | 0.9809 | 0.9713 | 0.9793 | 0.9611 |
|  | auROC | 0.9732 | 0.9773 | 0.9778 | 0.9715 | 0.9780 | 0.9780 | **0.9812** | 0.9718 | 0.9793 | 0.9622 |
|  | auPR | 0.9831 | 0.9855 | 0.9862 | 0.9822 | 0.9869 | 0.9866 | **0.9890** | 0.9818 | 0.9878 | 0.9758 |
| lon_channel | acc | 0.9837 | 0.9800 | 0.9793 | 0.9776 | 0.9807 | 0.9726 | 0.9790 | 0.9760 | 0.9671 | 0.9621 |
|  | pre | 0.9834 | 0.9779 | 0.9740 | 0.9668 | 0.9688 | 0.9740 | 0.9663 | 0.9753 | 0.9587 | 0.9417 |
|  | rec | 0.9847 | 0.9822 | 0.9847 | 0.9880 | 0.9931 | 0.9715 | 0.9917 | 0.9765 | 0.9755 | 0.9814 |
|  | F1 | 0.9840 | 0.9800 | 0.9793 | 0.9772 | 0.9807 | 0.9726 | 0.9788 | 0.9758 | 0.9669 | 0.9610 |
|  | auROC | 0.9837 | 0.9802 | 0.9797 | 0.9778 | 0.9810 | 0.9728 | 0.9791 | 0.9763 | 0.9677 | 0.9622 |
|  | auPR | 0.9883 | 0.9857 | 0.9860 | 0.9857 | 0.9889 | 0.9793 | 0.9874 | 0.9822 | 0.9776 | 0.9761 |
| Enzyme | acc | 0.9957 | 0.9954 | 0.9964 | 0.9911 | 0.9952 | 0.9930 | 0.9932 | 0.9942 | 0.9956 | 0.9945 |
|  | pre | 0.9999 | 0.9999 | 0.9999 | 0.9948 | 0.9997 | 0.9986 | 0.9983 | 0.9999 | 0.9986 | 0.9976 |
|  | rec | 0.9915 | 0.9908 | 0.9928 | 0.9874 | 0.9908 | 0.9874 | 0.9880 | 0.9885 | 0.9924 | 0.9914 |
|  | F1 | 0.9957 | 0.9954 | 0.9964 | 0.9911 | 0.9952 | 0.9930 | 0.9931 | 0.9942 | 0.9955 | 0.9945 |
|  | auROC | 0.9957 | 0.9954 | 0.9964 | 0.9911 | 0.9952 | 0.9930 | **0.9932** | 0.9942 | 0.9956 | 0.9946 |
|  | auPR | 0.9979 | 0.9977 | 0.9982 | 0.9943 | 0.9975 | 0.9962 | **0.9962** | 0.9971 | 0.9975 | 0.9967 |
