# Supplementary material for: Prediction of drug target interaction based on under sampling strategy and random forest algorithm
Source: PLoS One. 2025 Mar 6;20(3):e0318420. doi: 10.1371/journal.pone.0318420 (PMC11884685; doi:10.1371/journal.pone.0318420)
Supplement: S3 Table — (DOCX) [file pone.0318420.s003.docx]

| **Dataset** | **Metrics** | **Number of trees** | | | | | | | | | |
| --- | --- | --- | --- | --- | --- | --- | --- | --- | --- | --- | --- |
|  |  | 1000 | 900 | 800 | 700 | 600 | 500 | 400 | 300 | 200 | 100 |
| Nuclear_receptor | acc | 0.9167 | 0.8722 | 0.8712 | 0.8722 | 0.8667 | 0.8889 | 0.8833 | 0.8722 | 0.8722 | 0.8889 |
|  | pre | 0.8755 | 0.8783 | 0.8549 | 0.8692 | 0.8563 | 0.8817 | 0.8785 | 0.8529 | 0.8674 | 0.8914 |
|  | rec | 0.9639 | 0.8806 | 0.8973 | 0.8834 | 0.8847 | 0.9133 | 0.8967 | 0.8967 | 0.8901 | 0.8944 |
|  | F1 | 0.9118 | 0.8723 | 0.8664 | 0.8710 | 0.8612 | 0.8873 | 0.8776 | 0.8674 | 0.8710 | 0.8838 |
|  | **auROC** | **0.9231** | **0.8829** | **0.8828** | **0.8796** | **0.8785** | **0.9017** | **0.8970** | **0.8796** | **0.8793** | **0.9030** |
|  | **auPR** | **0.9530** | **0.9128** | **0.9150** | **0.9124** | **0.9094** | **0.9308** | **0.9209** | **0.9137** | **0.9148** | **0.9235** |
|  | Runtime(s) | 134.75 | 111.65 | 106.79 | 87.71 | 83.31 | 82.25 | 69.49 | 60.10 | 53.10 | 39.45 |
| GPCR | acc | 0.9803 | 0.9724 | 0.9772 | 0.9772 | 0.9780 | 0.9772 | 0.9709 | 0.9764 | 0.9819 | 0.9732 |
|  | pre | 0.9684 | 0.9642 | 0.9601 | 0.9651 | 0.9672 | 0.9654 | 0.9548 | 0.9654 | 0.9654 | 0.9670 |
|  | rec | 0.9921 | 0.9809 | 0.9934 | 0.9885 | 0.9886 | 0.9887 | 0.9869 | 0.9877 | 0.9984 | 0.9793 |
|  | F1 | 0.9799 | 0.9723 | 0.9763 | 0.9763 | 0.9776 | 0.9768 | 0.9702 | 0.9761 | 0.9814 | 0.9730 |
|  | **auROC** | **0.9803** | **0.9726** | **0.9770** | **0.9773** | **0.9782** | **0.9772** | **0.9712** | **0.9763** | **0.9819** | **0.9733** |
|  | **auPR** | **0.9881** | **0.9856** | **0.9866** | **0.9855** | **0.9861** | **0.9857** | **0.9822** | **0.9852** | **0.9805** | **0.9814** |
| lon_channel | acc | 0.9760 | 0.9631 | 0.9682 | 0.9699 | 0.9780 | 0.9702 | 0.9709 | 0.9848 | 0.9726 | 0.9648 |
|  | pre | 0.9621 | 0.9694 | 0.9588 | 0.9629 | 0.9679 | 0.9693 | 0.9612 | 0.9759 | 0.9563 | 0.9623 |
|  | rec | 0.9893 | 0.9575 | 0.9776 | 0.9769 | 0.9815 | 0.9712 | 0.9806 | 0.9936 | 0.9887 | 0.9670 |
|  | F1 | 0.9755 | 0.9632 | 0.9679 | 0.9697 | 0.9746 | 0.9701 | 0.9707 | 0.9846 | 0.9722 | 0.9645 |
|  | **auROC** | **0.9761** | **0.9637** | **0.9688** | **0.9705** | **0.9747** | **0.9707** | **0.9712** | **0.9850** | **0.9728** | **0.9651** |
|  | **auPR** | **0.9852** | **0.9712** | **0.9787** | **0.9794** | **0.9828** | **0.9780** | **0.9807** | **0.9908** | **0.9835** | **0.9741** |
| Enzyme | acc | 0.9803 | 0.9724 | 0.9772 | 0.9772 | 0.9780 | 0.9772 | 0.9709 | 0.9764 | 0.9819 | 0.9732 |
|  | pre | 0.9684 | 0.9642 | 0.9601 | 0.9651 | 0.9672 | 0.9654 | 0.9548 | 0.9654 | 0.9654 | 0.9670 |
|  | rec | 0.9921 | 0.9809 | 0.9934 | 0.9885 | 0.9886 | 0.9887 | 0.9869 | 0.9877 | 0.9984 | 0.9793 |
|  | F1 | 0.9799 | 0.9723 | 0.9763 | 0.9763 | 0.9776 | 0.9768 | 0.9702 | 0.9761 | 0.9814 | 0.9730 |
|  | **auROC** | **0.9803** | **0.9726** | **0.9770** | **0.9773** | **0.9782** | **0.9772** | **0.9712** | **0.9763** | **0.9819** | **0.9733** |
|  | **auPR** | **0.9881** | **0.9856** | **0.9866** | **0.9855** | **0.9861** | **0.9857** | **0.9822** | **0.9852** | **0.9805** | **0.9814** |
