# Supplementary material for: Prediction of drug target interaction based on under sampling strategy and random forest algorithm
Source: PLoS One. 2025 Mar 6;20(3):e0318420. doi: 10.1371/journal.pone.0318420 (PMC11884685; doi:10.1371/journal.pone.0318420)
Supplement: S4 Table — (DOCX) [file pone.0318420.s004.docx]

| **Dataset** | **Metrics** | **Max depth** | | | | | | | | | |
| --- | --- | --- | --- | --- | --- | --- | --- | --- | --- | --- | --- |
|  |  | 90 | 80 | 70 | 60 | 50 | 40 | 30 | 20 | 10 | None |
| Nuclear_receptor | acc | 0.8889 | 0.8944 | 0.8889 | 0.8853 | 0.8833 | 0.8889 | 0.8833 | 0.8833 | 0.8778 | 0.9167 |
|  | pre | 0.8783 | 0.8985 | 0.8686 | 0.8894 | 0.8567 | 0.8470 | 0.8985 | 0.8817 | 0.8692 | 0.8755 |
|  | rec | 0.9064 | 0.9062 | 0.9175 | 0.8900 | 0.9099 | 0.9417 | 0.8789 | 0.9015 | 0.8967 | 0.9639 |
|  | F1 | 0.8864 | 0.8980 | 0.8824 | 0.8879 | 0.8753 | 0.8797 | 0.8844 | 0.8808 | 0.8756 | 0.9118 |
|  | **auROC** | **0.8980** | **0.9017** | **0.9007** | **0.8845** | **0.8940** | **0.9005** | **0.8844** | **0.8968** | **0.8887** | **0.9231** |
|  | **auPR** | **0.9257** | **0.9301** | **0.9291** | **0.9202** | **0.9222** | **0.9360** | **0.9165** | **0.9249** | **0.9190** | **0.9530** |
| GPCR | acc | 0.9740 | 0.9709 | 0.9756 | 0.9732 | 0.9787 | 0.9724 | 0.9787 | 0.9701 | 0.9756 | 0.9803 |
|  | pre | 0.9654 | 0.9531 | 0.9626 | 0.9588 | 0.9623 | 0.9587 | 0.9699 | 0.9624 | 0.9654 | 0.9684 |
|  | rec | 0.9822 | 0.9882 | 0.9887 | 0.9874 | 0.9949 | 0.9859 | 0.9872 | 0.9777 | 0.9854 | 0.9921 |
|  | F1 | 0.9736 | 0.9701 | 0.9753 | 0.9726 | 0.9782 | 0.9720 | 0.9784 | 0.9697 | 0.9752 | 0.9799 |
|  | auROC | **0.9741** | **0.9712** | **0.9758** | **0.9730** | **0.9789** | **0.9721** | **0.9786** | **0.9702** | **0.9757** | **0.9803** |
|  | auPR | **0.9825** | **0.9825** | **0.9851** | **0.9833** | **0.9880** | **0.9825** | **0.9860** | **0.9795** | **0.9840** | **0.9881** |
| lon_channel | acc | 0.9682 | 0.9699 | 0.9655 | 0.9702 | 0.9692 | 0.9732 | 0.9638 | 0.9763 | 0.9760 | 0.9760 |
|  | pre | 0.9705 | 0.9658 | 0.9628 | 0.9643 | 0.9782 | 0.9747 | 0.9633 | 0.9794 | 0.9596 | 0.9621 |
|  | rec | 0.9659 | 0.9742 | 0.9687 | 0.9760 | 0.9615 | 0.9721 | 0.9648 | 0.9738 | 0.9925 | 0.9893 |
|  | F1 | 0.9681 | 0.9699 | 0.9655 | 0.9699 | 0.9696 | 0.9733 | 0.9639 | 0.9765 | 0.9757 | 0.9755 |
|  | auROC | **0.9685** | **0.9698** | **0.9658** | **0.9705** | **0.9693** | **0.9735** | **0.9640** | **0.9764** | **0.9760** | **0.9761** |
|  | auPR | **0.9757** | **0.9786** | **0.9752** | **0.9792** | **0.9754** | **0.9798** | **0.9734** | **0.9819** | **0.9842** | **0.9852** |
| Enzyme | acc | 0.9863 | 0.9892 | 0.9874 | 0.9927 | 0.9911 | 0.9949 | 0.9930 | 0.9894 | 0.9892 | 0.9927 |
|  | pre | 0.9861 | 0.9921 | 0.9952 | 0.9976 | 0.9966 | 0.9935 | 0.9983 | 0.9976 | 0.9993 | 0.9987 |
|  | rec | 0.9862 | 0.9863 | 0.9797 | 0.9877 | 0.9857 | 0.9951 | 0.9858 | 0.9815 | 0.9789 | 0.9867 |
|  | F1 | 0.9861 | 0.9892 | 0.9873 | 0.9927 | 0.9911 | 0.9948 | 0.9925 | 0.9894 | 0.9893 | 0.9927 |
|  | auROC | **0.9863** | **0.9893** | **0.9874** | **0.9927** | **0.9912** | **0.9949** | **0.9920** | **0.9894** | **0.9892** | **0.9927** |
|  | auPR | **0.9899** | **0.9927** | **0.9926** | **0.9957** | **0.9947** | **0.9959** | **0.9951** | **0.9941** | **0.9946** | **0.9960** |

^a^”None” means that the decision tree will not limit the depth of each subtree until the optimal model is found.
